# Nitrogen Removal Efficiency and Microbial Response Mechanism of *Hordeum vulgare* var. *coeleste* L. Straw as an External Carbon Source Under Different C/N Ratios

**DOI:** 10.3390/microorganisms14051024

**Published:** 2026-04-30

**Authors:** Renxu Wang, Yansong Wang, Yongchen Zong, Xiangyu Chen

**Affiliations:** 1 Research Center of Civil, Hydraulic and Power Engineering of Xizang, Department of Education of Xizang Autonomous Region, Xizang Agricultural and Animal Husbandry University, Linzhi 860000, China; 15611759752@163.com; 2 The College of Water Conservancy and Civil Engineering, Xizang Agricultural and Animal Husbandry University, No. 100 Yucai West Road, Bayi District, Linzhi 860000, China; wys1844974834@163.com (Y.W.); chenxiangyu@xza.edu.cn (X.C.)

**Keywords:** *Hordeum vulgare* var. *coeleste* straw, low C/N ratio, external carbon source, plateau area, microbial mechanism

## Abstract

To address the bottleneck of poor biological nitrogen removal efficiency caused by the extremely low carbon-to-nitrogen (C/N) ratio of domestic sewage in alpine plateau regions, this study used *Hordeum vulgare* var. *coeleste* L., a characteristic crop endemic to the Qinghai–Tibet Plateau, as raw material and adopted pretreated highland barley straw as an external carbon source. Three parallel experiments were carried out using the anaerobic–aerobic–anoxic sequencing batch reactor (AOA-SBR) process to investigate the nitrogen removal performance and functional succession of the microbial community in the AOA-SBR system under three C/N ratio ranges: 5~7, 7~9, and 9~11. The results showed that the addition of an external carbon source significantly improved nitrogen removal efficiency. The optimal C/N ratio range for nitrogen removal in this study was determined to be 7~9. A weakly alkaline environment was conducive to denitrification. The fermentation broth prepared by alkali pretreatment contained a large amount of readily biodegradable organic matter with low toxicity, and achieved excellent nitrogen removal performance, helping to realize cost reduction and efficiency improvement in wastewater treatment. At the optimal C/N ratio of 7~9, the average removal efficiencies of ammonia nitrogen (NH_4_^+^-N) and total nitrogen (TN) reached 94.46% and 61.32%, respectively, which were significantly improved compared with the blank control group without external carbon addition. During the experimental period, no obvious changes were observed in microbial abundance at the phylum level, whereas the community structure at the genus level responded significantly to the addition of a straw carbon source. Among them, genera with specific degradation capabilities for straw hydrolysates, such as *norank_f__Chitinophagaceae* and *unclassified_f__Comamonadaceae*, were highly sensitive to variations in the C/N ratio. These genera could partially replace the nitrification and denitrification functions of other microorganisms and played a key role in the nitrogen removal process. In contrast, *Thauera*, a typical conventional heterotrophic denitrifier, showed no significant response to changes in the C/N ratio, indicating that the straw-based external carbon source mainly affected microbial genera with specific hydrolysate-degrading functions.

## 1. Introduction

With the acceleration of urbanization in plateau areas, the discharge of domestic sewage has increased sharply, and the low carbon–nitrogen (C/N) ratio of sewage has become a prominent bottleneck restricting the efficiency of biological nitrogen removal in wastewater treatment plants (WWTPs) [1]. Xizang, as a typical alpine plateau region, is faced with dual adverse effects of low ambient water temperature and an extremely low C/N ratio of inlet water: low temperature inhibits the enzyme activity and metabolic rate of denitrifying microorganisms, while an insufficient carbon source limits the complete denitrification process, resulting in poor nitrogen removal efficiency of traditional biological treatment processes [2]. At present, WWTPs in Xizang mainly use sodium acetate and glucose as exogenous carbon sources to improve the C/N ratio, which leads to high operation costs and limits the sustainable development of wastewater treatment in the region [3,4,5,6].

Agricultural waste straw, as a low-cost and renewable biomass carbon source, has been widely studied in the field of wastewater denitrification [7,8]. Meanwhile, Xizang *Hordeum vulgare* var. *coeleste* was successfully selected for the National High-quality Agricultural Brand Cultivation Program in 2022. In 2025, the sown area of *Hordeum vulgare* var. *coeleste* in Xizang reached 2.33 million mu, indicating that *Hordeum vulgare* var. *coeleste* straw features distinct regional characteristics of Xizang and abundant raw material supplies. As the predominant agricultural product in Xizang, *Hordeum vulgare* var. *coeleste* achieves an annual output of 900,000 tons. A large quantity of its straw is either used as livestock feed or directly incinerated, which not only causes serious resource waste but also triggers prominent environmental pollution problems [9,10]. *Hordeum vulgare* var. *coeleste* straw is rich in lignocellulose, which can release soluble organic matter (such as acetic acid and lactic acid) through chemical pretreatment and anaerobic fermentation, and can be used as an exogenous carbon source to supplement the carbon demand for wastewater denitrification, realizing the resource utilization of agricultural waste [11,12]. However, research on the application of the *Hordeum vulgare* var. *coeleste* straw carbon source in plateau low C/N sewage denitrification is still lacking, and the response mechanism of microbial communities to the *Hordeum vulgare* var. *coeleste* straw carbon source under different C/N ratios in alpine environments has not been clarified [11,12].

In this study, three parallel anaerobic–aerobic–anoxic sequencing batch reactor (AOA-SBR) experiments were constructed with *Hordeum vulgare* var. *coeleste* straw as the exogenous carbon source, and the C/N ratios were set as 5~7, 7~9 and 9~11 to explore the optimal C/N interval for nitrogen removal in plateau sewage. Combined with 16S rRNA high-throughput sequencing technology, the changes in microbial community structure and the enrichment characteristics of functional bacteria under the action of a *Hordeum vulgare* var. *coeleste* straw carbon source were analyzed, and the key microbial taxa and their response mechanisms involved in the denitrification process were identified. The research results are expected to provide a new low-cost carbon source scheme for plateau low C/N sewage treatment, and lay a microbial theoretical foundation for the resource utilization of *Hordeum vulgare* var. *coeleste* straw in the field of environmental engineering.

## 2. Materials and Methods

### 2.1. Experimental Setup

Three parallel groups of AOA-SBR process experiments were conducted in this study. The reactor was a cylindrical barrel with an inner diameter of 19 cm and a height of 25 cm, and the effective volume was 5.67 L. The hydraulic retention time (HRT) was 12 h, and the temperature was controlled at 25 ± 1 °C. In this experiment, the operating water temperature was controlled at 25 °C. The reason was that large diurnal temperature variations (up to 10 °C) were observed in the study area during the preliminary experiment, which severely affected the experimental operation. To ensure stable performance of the experiment and in combination with relevant studies, 25 °C was determined to be suitable for wastewater treatment. The AOA operation mode was adopted as follows: influent for 0.5 h, anaerobic stirring for 2 h, aerobic aeration for 6 h, anoxic stirring for 2 h, sedimentation for 1 h, and effluent for 0.5 h. The volumetric exchange ratio was 50% per cycle and 50% per day, with a sludge discharge volume of 280 mL. The dissolved oxygen (DO) during the aeration phase was controlled at 3~5 mg·L^−1^, the mixed liquor suspended solids (MLSS) at 3000~5000 mg·L^−1^, and the sludge retention time (SRT) at 20 days. The sludge was taken from the Baji Wastewater Treatment Plant in Nyingchi City, Tibet, and acclimated with domestic sewage from our university for 20 days before the experiment.

This study investigated the effects of adding *Hordeum vulgare* var. *coeleste* straw as a carbon source on nitrogen and phosphorus removal under different carbon-to-nitrogen ratios (C/N). Reactor S1 served as the control group and was fed only with domestic sewage. Reactor S2 was fed with a mixture of unpretreated *Hordeum vulgare* var. *coeleste* straw fermentation broth and sewage (this group only provided graphical data without detailed investigation, as the C/N ratio of the unpretreated mixture made it difficult to reach the preset range). Reactor S3 was fed with a mixture of *Hordeum vulgare* var. *coeleste* straw fermentation broth pretreated with 2% (calculated by mass fraction) sulfuric acid and sewage. Reactor S4 was fed with a mixture of *Hordeum vulgare* var. *coeleste* straw fermentation broth pretreated with 2% (calculated by mass fraction) sodium hydroxide and sewage. Based on field surveys of the actual operation of more than 10 wastewater treatment plants in Xizang, including two A^2^/O process plants in Lhasa, one A^2^/O process plant in Shannan, two A^2^/O process plants and one MBR process plant in Xigaze, one multi-stage AO process plant in Nyingchi, one A^2^/O process plant combined with constructed wetland in Ngari, one A^2^/O process plant in Nagqu, and one A^2^/O process plant in Qamdo, it was found that the carbon-to-nitrogen (C/N) ratio of urban domestic sewage in Xizang is relatively low, and external carbon sources are commonly used in these wastewater treatment plants. Relevant studies have shown that a C/N ratio between 5 and 8 can significantly improve nitrogen removal efficiency, but this range is relatively broad. Therefore, this experiment designed three more precise intervals, including a range higher than 5~8, to verify the effect of increasing the C/N ratio on nitrogen removal performance. Through preliminary experiments, a comparative study was carried out by controlling the C/N ratio within three ranges: 5~7, 7~9, and 9~11.

**Figure 1:**
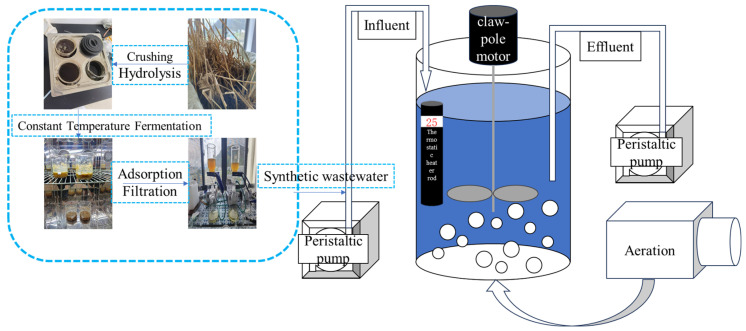
Installation operation diagram.

The external carbon source was prepared as follows: *Hordeum vulgare* var. *coeleste* straw was collected from the experimental farm of the College of Horticulture and Plant Science at our university. The collected straw was cut into 2~4 cm pieces, rinsed twice with deionized water, dried, and then ground into 60-mesh powder using a grinder. A total of 20 g of the straw powder was placed into a 1 L beaker, to which 1000 mL of 2% (mass fraction) sulfuric acid (H_2_SO_4_) solution and 1000 mL of 2% (mass fraction) sodium hydroxide (NaOH) solution were added separately [7,8]. Hydrolysis was performed in a 90°C water bath with continuous stirring for 1 h. After cooling, the pH was adjusted to neutral, and the beaker was sealed with plastic wrap and fermented at a constant temperature of 30°C for 12 h. The supernatant was collected from the fermentation broth via vacuum filtration and used as the *Hordeum vulgare* var. *coeleste* straw-based external carbon source. Gas chromatography–mass spectrometry (GC-MS) was employed to analyze the components of the fermentation broth, and microbial inhibitors and their removal methods were identified based on the compositional results. Figure 1 illustrates the preparation process of the highland barley straw carbon source and the reactor operation procedure.

### 2.2. Water Quality Detection Methods

The influent carbon-to-nitrogen (C/N) ratios of the external carbon source reactors S3 and S4 were controlled within three ranges: 5~7, 7~9, and 9~11. These relatively precise C/N intervals were adopted to investigate the effect of external carbon source addition on nitrogen removal. The experiment was continuously operated and monitored for 150 days, with water quality measured every two days. Twenty-five datasets were collected for each interval, yielding a total of 75 water quality datasets to ensure the representativeness of the experimental results. These data were used to analyze the impacts of external carbon source addition on nitrogen and phosphorus removal efficiency as well as microbial genus composition. In this study, a Shengaohua 6B-3000A water quality analyzer (official website: https://www.sahkeji.com, accessed on 3 April 2026) was used to determine the following indicators: chemical oxygen demand (COD, potassium dichromate method), ammonia nitrogen (NH_4_^+^-N, Nessler’s reagent spectrophotometry), total nitrogen (TN, potassium persulfate UV spectrophotometry), nitrate nitrogen (NO_3_^−^-N, UV spectrophotometry), and nitrite nitrogen (NO_2_^−^-N, N-(1-naphthyl)ethylenediamine spectrophotometry). A Hach HQ40d portable meter (official website: https://www.hach.com.cn/, accessed on 3 April 2026) was employed to measure environmental parameters including temperature (T), dissolved oxygen (DO), and pH value.

### 2.3. Component Analysis of Fermentation Broth

The chemical compositions of acid-pretreated and alkali-pretreated fermentation broths were determined by gas chromatography–mass spectrometry (GC-MS) using a Shimadzu GCMS-QP2010 SE system (Shimadzu Corporation, Kyoto, Japan). The chromatographic conditions were as follows: HP-5MS capillary column (30 m × 0.25 mm × 0.25 μm); injector temperature, 250 °C; carrier gas, helium (purity ≥ 99.999%); column flow rate, 1.0 mL/min; split ratio, 10:1; and temperature programming: initial temperature of 50 °C maintained for 2 min, then ramped to 250 °C at a rate of 10 °C/min and held for 5 min. The mass spectrometric conditions were as follows: electron impact ionization (EI) source; electron energy, 70 eV; ion source temperature, 230 °C; transfer line temperature, 250 °C; mass scan range, 35~450 *m*/*z*.

The obtained mass spectra were compared with the NIST 20 mass spectral library for compound identification. The relative content of each component was calculated by the peak area normalization method. Based on the biodegradability characteristics reported in the literature, the identified components were divided into four categories: rapidly biodegradable carbon sources (volatile fatty acids, ketones, alcohols with carbon chain length ≤ 8), slowly biodegradable carbon sources (organic compounds with carbon chain length 9~16), non-biodegradable substances (aromatic hydrocarbons and lignin derivatives), and toxic/inhibitory substances (furfural, hydroxymethylfurfural, and phenolic compounds).

### 2.4. Microbial Detection

A total of 36 microbial samples were collected in this study. The initial sludge sample was obtained from the laboratory sludge culture device, and the remaining microbial samples were collected from each reactor every 16 days and stored at −80 °C (model: Meiling DW-HL528). The 36 activated sludge microbial samples collected from the SBR reactors in this experiment, which were stored at −80 °C, were entrusted to Majorbio Bio-Pharm Technology Co., Ltd. (Shanghai, China) (https://www.majorbio.com) for detection. Raw paired-end sequencing reads were subjected to quality control using fastp [13] (https://github.com/OpenGene/fastp, version 0.23.4) and merged using FLASH [14] (https://ccb.jhu.edu/software/FLASH/index.shtml, version 1.2.11) according to the following criteria: Bases with a quality score below 20 at the 3′ end of reads were trimmed. A sliding window of 50 bp was set, and bases downstream were truncated if the average quality score within the window was less than 20. Reads shorter than 50 bp after quality control were discarded, and reads containing more than 5 ambiguous bases (N) were removed. Paired-end (PE) reads were merged into single sequences based on their overlap, with a minimum overlap length of 10 bp. The maximum allowed mismatch ratio in the overlapping region was 0.2, and sequences failing this criterion were filtered out. Samples were demultiplexed and sequence orientation was adjusted according to barcodes and primers at both ends. Zero mismatches were allowed for barcodes, and a maximum of 2 mismatches were allowed for primers.

The quality-controlled and merged sequences were clustered into operational taxonomic units (OTUs) at a 97% sequence identity threshold using USEARCH [15,16] (http://drive5.com/usearch/, version 11), and chimeric sequences were removed from the OTUs. Taxonomic annotation of OTUs was performed by alignment against the SILVA138.2/16S_Bacteria database using the RDP Classifier [17] (https://ngdc.cncb.ac.cn/databasecommons/database/id/237, version 2.11) with a confidence threshold of 70%. To minimize the influence of sequencing depth on subsequent data analysis, the abundance data of the three samples were subjected to rarefaction. Alpha diversity indices and beta diversity analysis were calculated using Mothur (version 1.30.2) and visualized with R software (version 3.3.1). Principal coordinate analysis (PCoA) based on Bray–Curtis distances combined with ANOSIM/Adonis methods was used to evaluate the overall differences in bacterial communities among samples. Bar charts and heatmaps of community composition were applied to visually display the distribution of dominant species.

Significant differences between groups were assessed using the Kruskal–Wallis test (for multiple groups) or Wilcoxon test (for two groups) in R. Furthermore, LEfSe analysis was used to identify key species contributing to inter-group differences. Correlation analysis between microbial species and physicochemical parameters was conducted to screen for significantly correlated species. Random forest machine learning was then employed based on these candidate species to identify biomarkers capable of significantly distinguishing different groups. Redundancy analysis (RDA) and canonical correspondence analysis (CCA) were used to investigate the relationships between microbial communities and physicochemical factors. Variation partitioning analysis (VPA) was performed using the “vegan” package in R (version 3.3.1) to identify the main environmental factors shaping microbial communities. For functional prediction, 16S rRNA gene-based functional analysis was carried out using PICRUSt2 [18] (version 2.2.0). All data analyses were completed on the Majorbio Cloud Platform (https://v.majorbio.com).

## 3. Results

### 3.1. Water Quality Indexes

**Figure 2:**
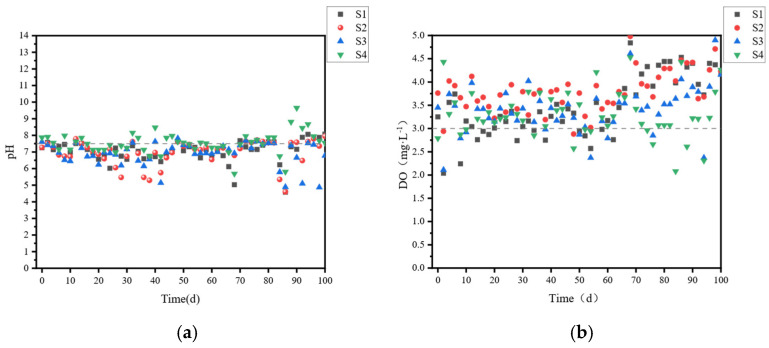
Variation diagrams of pH (**a**) and DO (**b**) for each unit.

Furthermore, as shown in Figure 2 (pH variation), the reactor environment of S4 remained slightly alkaline (pH ≈ 7.5~8.0) throughout most of the 100-day operation period after neutralization, which is more favorable for the growth and metabolic activity of denitrifying bacteria compared to the slightly acidic environment (pH occasionally dropped below 6.0) observed in S3. This pH difference also contributed to the superior nitrogen removal performance of the alkali-pretreated group [19,20,21]. Notably, COD and NH_4_^+^-N were efficiently removed in all reactors throughout the experiment, with average removal rates ranging from 32.04% to 83.34% and 52.80% to 94.46%, respectively. The removal efficiencies of these two pollutants increased significantly with external carbon source addition, but the differences between the acid-pretreated (S3) and alkali-pretreated (S4) groups were relatively small. In contrast, TN removal was the main limiting factor for the system’s treatment performance [22], showing the most pronounced differences among different carbon source types and C/N ratios, with average removal rates ranging from 28.83% to 61.32% across all operating conditions.

**Figure 3:**
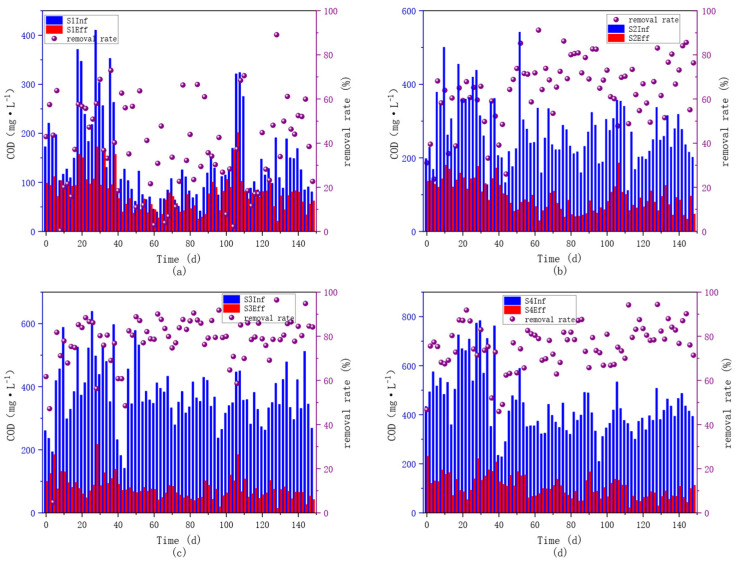

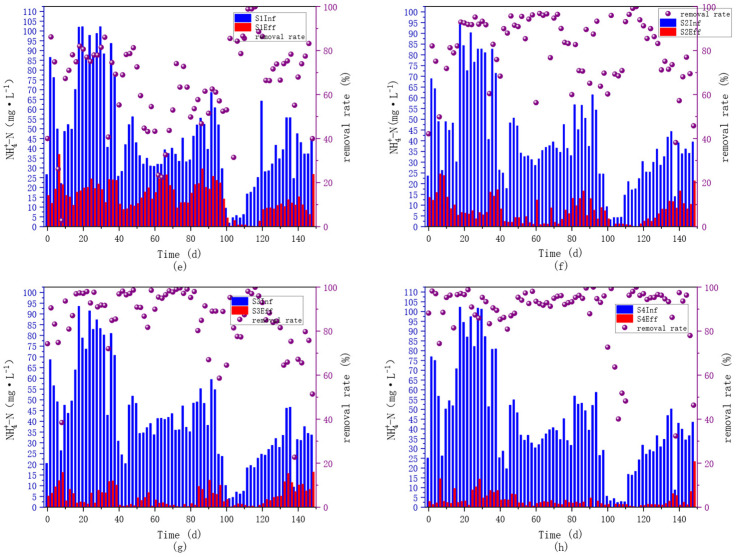

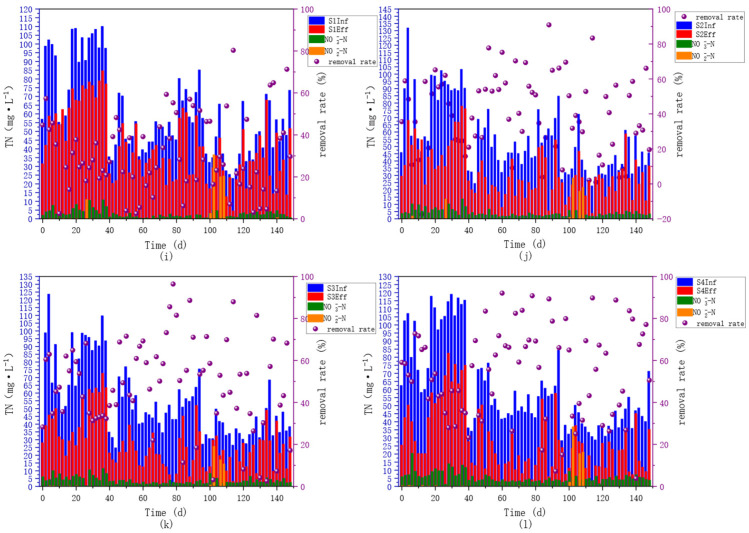
Pollutant removal efficiency diagram for each unit. ((**a**): Influent and effluent COD concentrations and removal efficiency in Reactor S1. (**b**): Influent and effluent COD concentrations and removal efficiency in Reactor S2. (**c**): Influent and effluent COD concentrations and removal efficiency in Reactor S3. (**d**): Influent and effluent COD concentrations and removal efficiency in Reactor S4. (**e**): Influent and effluent NH_4_^+^-N concentrations and removal efficiency in Reactor S1. (**f**): Influent and effluent NH_4_^+^-N concentrations and removal efficiency in Reactor S2. (**g**): Influent and effluent NH_4_^+^-N concentrations and removal efficiency in Reactor S3. (**h**): Influent and effluent NH_4_^+^-N concentrations and removal efficiency in Reactor S4. (**i**): Influent and effluent TN concentrations and removal efficiency in Reactor S1. (**j**): Influent and effluent TN concentrations and removal efficiency in Reactor S2. (**k**): Influent and effluent TN concentrations and removal efficiency in Reactor S3. (**l**): Influent and effluent TN concentrations and removal efficiency in Reactor S4).

According to the comprehensive analysis of Figure 3 (COD and NH_4_^+^-N removal) and Figure 3 (TN removal), reactors supplemented with external carbon sources exhibited significantly better nitrogen removal performance than the blank control group (S1), and the reactor dosed with alkali-pretreated *Hordeum vulgare* var. *coeleste* straw hydrolysate (S4) achieved the optimal and most stable treatment effect. This confirms that external carbon source addition can effectively compensate for the carbon deficiency in low C/N sewage and enhance the biological nitrogen removal efficiency of the system. The nitrogen removal efficiency of all reactors showed a consistent trend of first increasing and then decreasing with the increase in influent C/N ratio, and the best performance was obtained at the C/N range of 7~9. Specifically, as shown in the Table 1, at C/N = 7~9, the average TN removal rates were 30.84% (S1, blank), 49.36% (S2, untreated straw), 58.46% (S3, acid-pretreated), and 61.32% (S4, alkali-pretreated), respectively, which were 6.98~13.98% higher than those at C/N = 5~7 and 11.14~20.20% higher than those at C/N = 9~11. This finding is consistent with previous studies reporting that the optimal C/N ratio for conventional biological denitrification is typically 5~8, indicating that excessive carbon source addition does not further improve treatment performance but may cause secondary COD pollution [3,23]. Among the experimental groups with different pretreatment methods, the S4 group (alkali-pretreated) consistently outperformed the S3 group (acid-pretreated) at the same C/N level, even though the influent C/N ratios of S3 and S4 were strictly controlled to be identical by adjusting the mixing ratio of fermentation broth and raw sewage during water preparation. At the optimal C/N = 7~9, the TN removal efficiency of S3 was 2.86% lower than that of S4, and its removal performance showed more obvious fluctuations as observed from the time-series curves in Figure 3. This performance discrepancy can be attributed to two key factors: first, acid pretreatment tends to generate more refractory multi-chain soluble organic compounds in the hydrolysate, which cannot be rapidly utilized by denitrifying bacteria; second, although both hydrolysates were neutralized before anaerobic fermentation, alkali pretreatment promoted more thorough degradation of macromolecular cellulose and hemicellulose into readily biodegradable volatile fatty acids (VFAs), providing a more sufficient and sustainable carbon source for the denitrification process.

### 3.2. Component Analysis of Hordeum vulgare var. coeleste Straw Fermentation Broth

**Figure 4:**
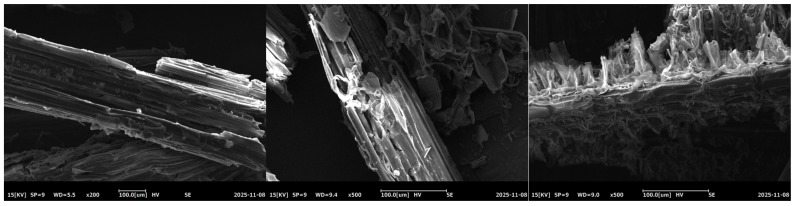
Scanning electron microscopy.

According to the scanning electron microscopy (SEM) results shown in Figure 4, the surface of untreated *Hordeum vulgare* var. *coeleste* straw exhibited longitudinal textures and grooves without obvious damage, retaining the macroscopic morphology of natural fibers. For acid-pretreated straw, the fibers were twisted and aggregated, with local bending, cracking and layering, as well as fiber deformation and breakage, reflecting a mechanical characteristic of both flexibility and brittleness.

Alkali pretreatment caused severe damage to the surface of highland barley straw powder. Fiber bundles were fractured, short columnar fibers were arranged with vertical cross-sections, and fibers were shortened, showing a fractured cross-sectional structure. Alkali treatment altered the porosity and permeability of highland barley straw. This is consistent with the conclusion that alkali-pretreated highland barley straw releases more carbon sources, and explains from a microscopic perspective why alkali pretreatment achieves higher carbon release efficiency than other treatment methods.

**Figure 5:**
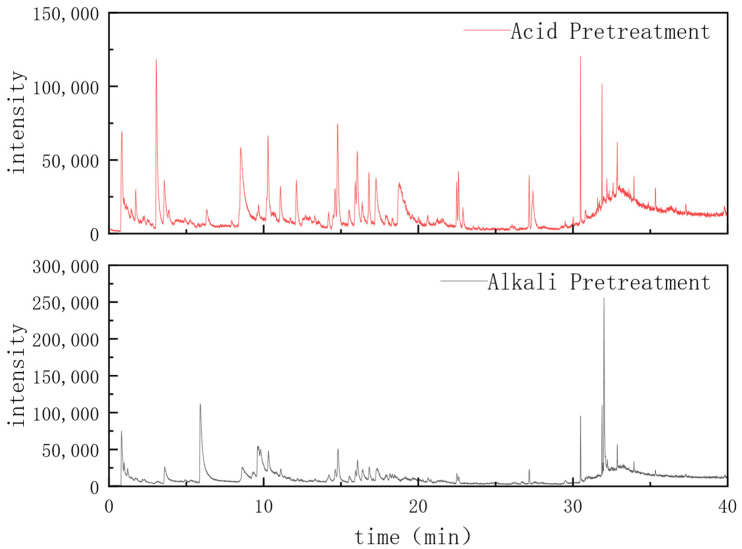
Chromatography–mass spectrometry.

As can be seen from the mass spectrometry and chromatography detection report in Figure 5, the top 100 peaks were summarized, and the contents of the detected substances were calculated based on peak areas against the spectral library, followed by the statistical summary of the main components. As can be seen from Table 2, acid treatment releases a relatively large amount of carbon source, with COD concentration ranging from 3000 mg·L^−1^ to 4000 mg·L^−1^. The composition is complex: only 25% is a rapidly biodegradable carbon source, 40~50% is biodegradable but degrades slowly, and toxic substances account for 20%. The high proportion of inhibitory substances results in inferior performance compared with alkali-treated fermentation broth under the same conditions. Alkali treatment yields the highest carbon content, with COD concentration exceeding 8000 mg·L^−1^, although part of the carbon source is difficult to utilize (which may explain the relatively high effluent COD concentration in Reactor S4). Only three types of high-quality rapidly biodegradable carbon sources are present, namely 2-Heptanone, 2-Octanone, and 3-Octanone, accounting for approximately 30%. Biodegradable but slowly degrading carbon sources account for about 30%, non-biodegradable substances about 20%, toxic and inhibitory substances about 10%, and other substances about 10%. Compared with acid-treated and untreated fermentation broths, alkali-treated fermentation broth features a higher carbon yield, a higher proportion of utilizable carbon sources, and lower toxic and inhibitory effects [24,25]. Prolonging fermentation time or applying adsorption treatment can be considered to reduce toxic inhibition.

### 3.3. Microbial Community

**Figure 6:**
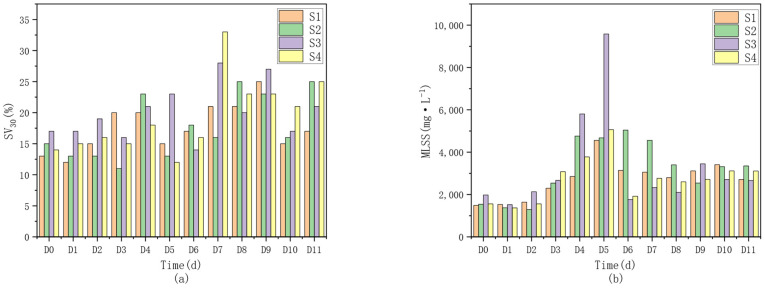
Variation diagrams of SV30 (**a**) and MLSS (**b**) for each unit.

It can be seen from Figure 6 that the sludge concentration gradually increased with the progress of the experiment and then tended to stabilize. The MLSS concentration rose rapidly from an initial level of approximately 2000 mg·L^−1^ during D1–D5, reflecting that sludge bulking could occur in the short term when the C/N ratio was increased by external carbon source addition [26]. After the microorganisms adapted to the corresponding conditions, the sludge concentration became relatively stable. Sludge bulking in the initial operation stage may be attributed to the relatively stable environment and microbial community structure without an external carbon source during the initial sludge acclimation period. As the experiment proceeded, the addition of external carbon sources with different pretreatment methods altered the system environment, leading to corresponding changes in the microbial community. According to calculations, the sludge retention time (SRT) and sludge loading rate were as follows: For S1, SRT = 20 d, and sludge loading rate = 0.011~0.036 g·COD/(g·MLSS·d). For S3, SRT = 20 d, and sludge loading rate = 0.0209~0.1316 g·COD/(g·MLSS·d). For S4, SRT = 20 d, and sludge loading rate = 0.0494~0.1825 g·COD/(g·MLSS·d). The relatively long sludge age, low sludge loading rate, and small sludge discharge volume were caused by the low initial MLSS of only 2000 mg·L^−1^ in the early stage [27], with a daily sludge discharge of only 280 mL. The sludge discharge volume was reduced to avoid excessive sludge wasting that would negatively affect nitrogen removal efficiency.

**Figure 7:**
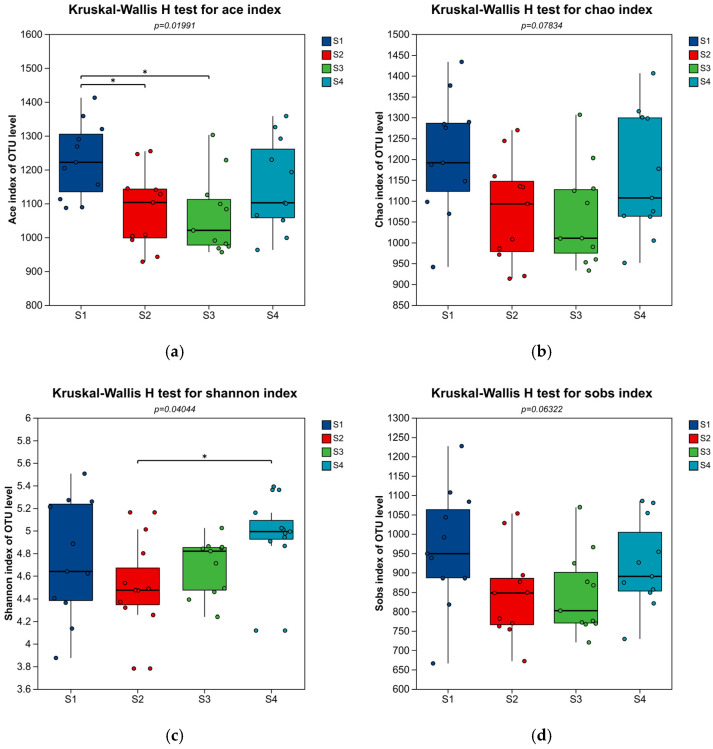
Alpha index inter-group difference test. * represents correlation, the *p*-value is given in the graph.

In this study, the Kruskal–Wallis H test (Figure 7) was used to analyze the inter-group differences in the Ace index among the three groups of samples, S1, S3, and S4. The results showed significant overall differences in the Ace index among the three groups (*p* = 0.01842), indicating that the grouping factor exerted a significant effect on the species richness of the community. Boxplot analysis revealed that the Ace index median of group S1 was the highest, corresponding to the highest level of species richness, while group S3 had the lowest median and the lowest species richness, with group S4 intermediate between the two. The microbial community in Reactor S1 exhibited the highest species richness, which was most favorable for the growth and reproduction of diverse microorganisms, accompanied by relatively high functional redundancy of the community. The species richness in Reactors S2 and S3 decreased significantly, indicating that the two treatment conditions exerted a strong directional selection effect on the microbial communities, eliminating a large number of maladapted species and resulting in a simplified community structure. The species richness in Reactor S4 was intermediate between those of S1, S2 and S3. However, it showed extremely high intra-group variability, suggesting poor stability of the microbial community under this treatment condition, yet its degradation capacity for complex pollutants and resistance to shock loading were stronger. In addition, the group showed extremely high intra-group variability, indicating either poor stability of the microbial community under these conditions or a lack of experimental reproducibility that requires further verification. This may be attributed to the fact that S1, as the control group fed with domestic sewage, exhibited better environmental adaptability than S3 and S4, whereas the addition of *Hordeum vulgare* var. *coeleste* straw carbon source in S3 and S4 exerted a strong selective effect on microorganisms, with the dominant genera mainly including *norank_f__Chitinophagaceae* and *unclassified_f__Comamonadaceae*, which showed strong adaptability to the external carbon source [28]. Pairwise comparisons between groups showed highly significant differences in the Ace index between S1 and S3 (*p* ≤ 0.01), whereas no significant differences were observed between S1 and S4 or between S3 and S4 (*p* > 0.05). No significant overall difference in the Chao index was detected among the three groups (*p* = 0.09896). The median Chao index of S1 remained the highest among the three groups, indicating the optimal species richness, while S3 had the lowest median and poorest species richness, with S4 intermediate and closer to S1. This trend was consistent with that of the Ace index. It is speculated that since S1 served as the control group with sewage only, its microbial community was not disturbed by the selective effect of the *Hordeum vulgare* var. *coeleste* straw carbon source, whereas the addition of such a carbon source in S3 and S4 led to the enrichment of dominant genera, resulting in overall lower species richness than S1. Despite numerical differences among groups, no statistically significant pairwise differences were found (*p* > 0.05). The inter-group difference patterns of the Shannon and Sobs indices were consistent with those mentioned above, and no obvious inter-group differences in species richness were observed. This also reflects that the addition of the *Hordeum vulgare* var. *coeleste* straw carbon source exerted little effect on microbial species richness without significant negative impacts, and improved nitrogen removal efficiency without compromising other functions of the microbial community. Furthermore, it was found that the addition of external carbon sources affected the microbial species diversity in reactors S2, S3 and S4, resulting in a decrease in their abundance compared with the control group S1. However, the reduction in microbial diversity does not indicate a decline in microbial nitrogen removal efficiency. On the contrary, the addition of external carbon sources promoted the enrichment of dominant genera, improved nitrogen removal efficiency, and led to the isolation of unique genera.

**Figure 8:**
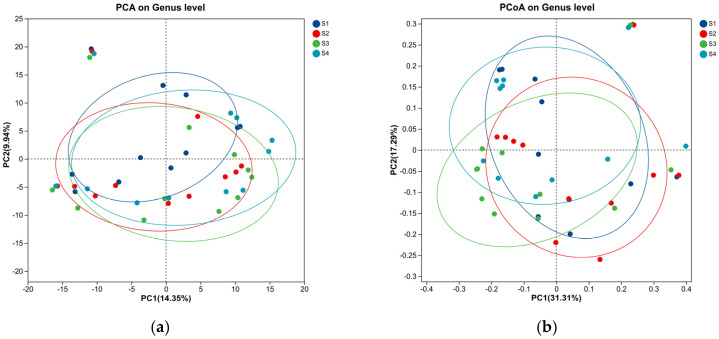
Beta diversity PCA (**a**) and PCoA (**b**) analysis.

In the principal component analysis (PCA, Figure 8) ordination plot based on genus-level microbial community composition, the first two principal components (PC1 and PC2) explained 14.35% and 9.94% of the total variance in community structure, respectively, with a cumulative explanatory rate of 25.67%. The 95% confidence ellipses clearly showed that samples from groups S1, S2, S3, and S4 formed relatively independent clusters, indicating that samples within the same group shared high similarity in microbial community structure, while distinct compositional differences existed between different treatment groups. Specifically, samples from the control group S1 were predominantly distributed in the negative region of PC1 and the medium-low region of PC2, whereas samples from group S4 were concentrated in the positive region of PC1 and the medium-high region of PC2. The spatial distance between S1 and S4 was the largest among all pairwise comparisons, with samples from groups S2 and S3 located in the transitional zone between them. This spatial distribution pattern suggested that the addition of the *Hordeum vulgare* var. *coeleste* straw carbon source induced significant differentiation in microbial community structure, and the degree of structural divergence was positively correlated with the carbon source dosage and operational conditions. Notably, group S4 exhibited the largest intra-group dispersion within its confidence ellipse, which was consistent with the high intra-group variability observed in the Alpha diversity analysis, further confirming the relatively unstable community state under this specific treatment condition.

Environmental factor loading analysis was performed to explore the correlations between key water quality parameters and microbial community differentiation. The length of the red arrows in the plot represents the contribution degree of each environmental factor to community variation, while the angle between two arrows indicates their correlation coefficient. The results revealed that total nitrogen (TN), ammonium nitrogen (NH_4_^+^-N), nitrate nitrogen (NO_3_^−^-N), total phosphorus (TP), and chemical oxygen demand (COD) were the dominant driving factors shaping the microbial community structure, showing strong positive correlations with the negative axis of PC1 and positive axis of PC2. In contrast, pH, carbon-to-nitrogen (C/N) ratio, and mixed liquor suspended solids (MLSS) made relatively weaker contributions to community differentiation. Among them, pH and C/N ratio were positively correlated with the positive axis of PC1, and pH also showed a weak positive correlation with the positive axis of PC2. Samples from group S4 were closely aligned with the directional arrows of C/N ratio and MLSS, indicating that the addition of the *Hordeum vulgare* var. *coeleste* straw carbon source was the primary determinant of the unique community characteristics in the S4 reactor. Samples from group S3 were distributed in the central region of the ordination plot and were jointly affected by multiple environmental factors without a single dominant driving factor, reflecting the transitional nature of its microbial community structure between the control group and the high-carbon-source group.

To further verify the above findings and overcome the limitations of PCA (which is based on Euclidean distance and assumes linear relationships), principal coordinate analysis (PCoA) was conducted based on the Bray–Curtis dissimilarity matrix, which is more suitable for analyzing microbial community data as it accounts for both species presence/absence and relative abundance. The PCoA results showed that the first two principal coordinates explained 31.31% and 17.29% of the total community structural variation, respectively, with a cumulative explanatory rate of 48.6%, which was significantly higher than that of PCA. Consistent with the PCA results, samples from different groups exhibited a clear trend of intra-group aggregation and inter-group separation along the coordinate axes. This robust pattern confirmed that the different treatment regimens significantly altered the genus-level microbial community structure, and each reactor developed relatively independent species composition characteristics adapted to its specific environmental conditions. For instance, the dominant genera in reactors S3 and S4, such as *norank_f__Chitinophagaceae* and *unclassified_f__Comamonadaceae*, were highly adapted to the environment supplemented with the *Hordeum vulgare* var. *coeleste* straw carbon source [28]. Meanwhile, several specific genera that were either absent or present at extremely low abundance in the control reactor S1 emerged and became enriched in the carbon-amended reactors, which directly contributed to the observed community structural differentiation. These changes in community composition were closely associated with the enhanced nitrogen removal performance observed in the carbon source addition groups, as these enriched genera are known to play crucial roles in denitrification processes.

**Figure 9:**
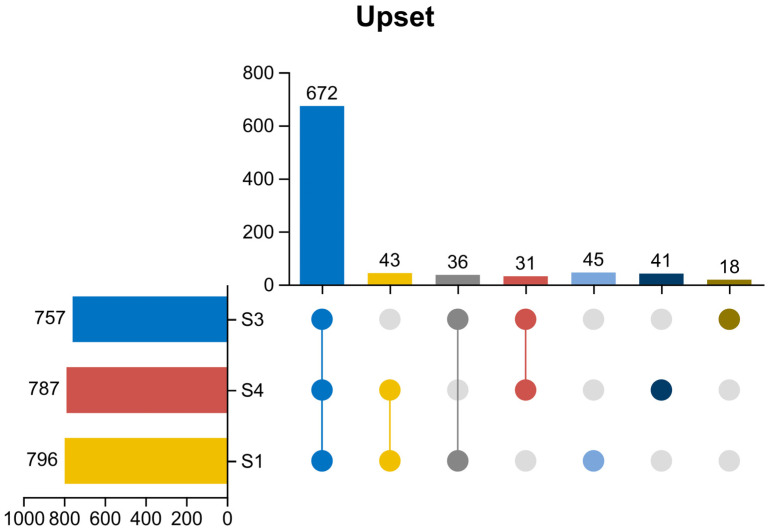
Species UpSet analysis.

As shown in Figure 9 (UpSet plot), the overlap and specificity of microbial species composition among the three reactors were analyzed to reveal the effects of different carbon source pretreatments on microbial community structure. The total number of detected species in reactors S1 (blank control), S4 (alkali-pretreated straw), and S3 (acid-pretreated straw) was 796, 787, and 757, respectively. A total of 672 core species were shared by all three groups, accounting for 84.4~88.8% of the total species in each reactor, indicating that the basic microbial community composition of the three systems was highly similar, with only minor species-specific differences. The number of species shared between any two groups ranged from 31 to 43: specifically, 43 species were shared by S1 and S4, 36 species by S1 and S3, and 31 species by S3 and S4. In terms of unique species, S1 harbored the largest number of unique species (45), followed by S4 (41) and S3 (18).

The highest species richness was observed in the blank control group (S1), reflecting that the original sewage environment caused minimal disturbance to the indigenous microorganisms and maintained a relatively high alpha diversity. In contrast, the species richness decreased in both S3 and S4 after external carbon source addition. This phenomenon suggests that carbon source supplementation exerted a selective pressure on the microbial community: only species capable of utilizing the added carbon sources could survive and proliferate, leading to the enrichment of functional dominant genera with significantly elevated relative abundances, which in turn contributed to the improved nitrogen and phosphorus removal performance of the systems. Notably, the species richness in S3 was significantly lower than that in S4. This discrepancy may be attributed to the stronger toxic effects of acid-pretreated *Hordeum vulgare* var. *coeleste* straw hydrolysate on microorganisms. As mentioned earlier, acid pretreatment tends to generate more refractory organic compounds and potential toxic byproducts, which inhibit the growth of some sensitive microbial species. This finding is consistent with the previous result that the nitrogen and phosphorus removal efficiency of S4 was significantly superior to that of S3 at the same C/N ratio.

**Figure 10:**
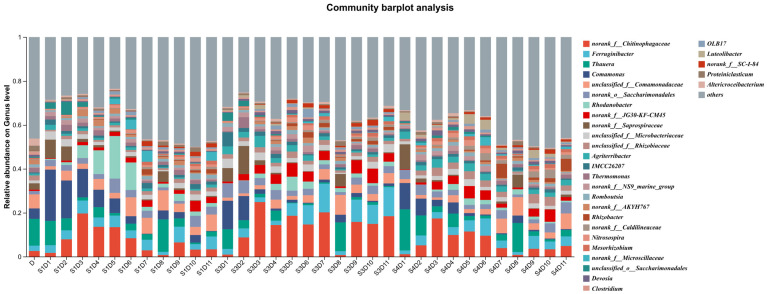
Microbial abundance at the genus level.

As shown in Figure 10 (genus-level community barplot), external carbon source addition significantly reshaped the microbial community structure at the genus level, and the inter-group differences became increasingly pronounced with experimental duration. All three reactors exhibited consistent temporal succession: the early stage dominant genera *(Comamonas, Thauera*) were gradually replaced by *norank_f__Chitinophagaceae* as the absolute dominant genus in the late operation stage.

*norank_f__Chitinophagaceae* (phylum *Bacteroidota*), a core functional genus specialized in degrading complex polysaccharides, became the most abundant genus in all reactors after Day 6. Notably, its relative abundance reached >40% in the late stage of the S3 group (acid-pretreated straw), which was significantly higher than that in S1 (30~35%) and S4 (32~37%). Chitin, also known as poly-N-acetylglucosamine, is a nitrogen-containing polysaccharide (carbohydrate polymer). It is a long chain composed of N-acetylglucosamine monomers linked by chemical bonds, which can be simply understood as amino-modified cellulose [28]. This genus was universally enriched in all carbon-added groups, directly reflecting its positive response to the input of straw-derived organic matter.

*Comamonas* (phylum *Proteobacteria*), a well-characterized denitrifying bacterium capable of degrading aromatic compounds, was the dominant genus in the inoculated sludge (D) and the early stage of S1 (D1~D5), with a relative abundance of 20~40%. However, its abundance sharply decreased to <10% in the late stage of all reactors, especially in the blank control group (S1) without external carbon supplementation [20,21]. Similarly, *unclassified_f__Comamonadaceae*, another denitrifying genus belonging to Proteobacteria, also showed a significant downward trend [29,30]. This genus is highly sensitive to environmental perturbations; the drastic changes in system redox conditions and carbon source composition caused by external carbon addition may have inhibited its growth, indirectly affecting the nitrogen removal efficiency [5,31,32].

*Thauera* (phylum Proteobacteria), a key heterotrophic denitrifier that reduces nitrate to nitrogen gas and degrades aromatic hydrocarbons, exhibited a “first decrease then increase” abundance trend throughout the experiment. Its relative abundance was 15~20% in the early stage of S1 and maintained at 5~10% in the late stage of all three reactors. Remarkably, *Thauera* retained a relatively high abundance even in the carbon-deficient S1 group, indicating that its denitrification activity is not strictly limited by the C/N ratio, which is consistent with previous reports [33,34,35].

*norank_f__Saprospiraceae* (phylum *Bacteroidota*), a filamentous bacterium with nitrification, phosphorus removal and protein degradation capabilities, showed a characteristic dynamic pattern of “low in acclimation, elevated in mid-stage, and declined in late stage” [36,37]. Its abundance was extremely low in the sludge acclimation phase (D) due to the low organic load of domestic sewage. After formal operation, the increased influent COD concentration induced the rapid proliferation of filamentous bacteria, which corresponded exactly to the temporary sludge bulking observed in D1~D5. As the microbial community gradually adapted to the high-carbon environment, the abundance of *norank_f__Saprospiraceae* decreased significantly in the late stage, consistent with the stabilization of sludge concentration.

Comparing the three groups, the S3 community showed the highest degree of simplification, with *norank_f__Chitinophagaceae* being excessively enriched and the abundance of other functional genera being suppressed. In contrast, the S4 group (alkali-pretreated straw) maintained a more balanced community structure with higher species evenness, which was more similar to that of the S1 group. This difference can be attributed to the milder selective pressure exerted by alkali-pretreated hydrolysate: as previously demonstrated, acid-pretreated hydrolysate contains more refractory macromolecular compounds and toxic byproducts, which eliminate most sensitive microorganisms and lead to the overgrowth of a few tolerant genera. This result is fully consistent with the UpSet analysis showing that S3 had the lowest species richness, and also explains why the nitrogen and phosphorus removal performance of S4 was more stable and superior to that of S3.

Overall, external carbon source addition reshaped the microbial community by selecting for straw-utilizing functional genera. Alkali-pretreated *Hordeum vulgare* var. *coeleste* straw hydrolysate not only provided sufficient biodegradable carbon for denitrification but also preserved a more diverse and resilient microbial community, which is beneficial for the long-term stable operation of low C/N wastewater treatment systems.

## 4. Discussion

**Figure 11:**
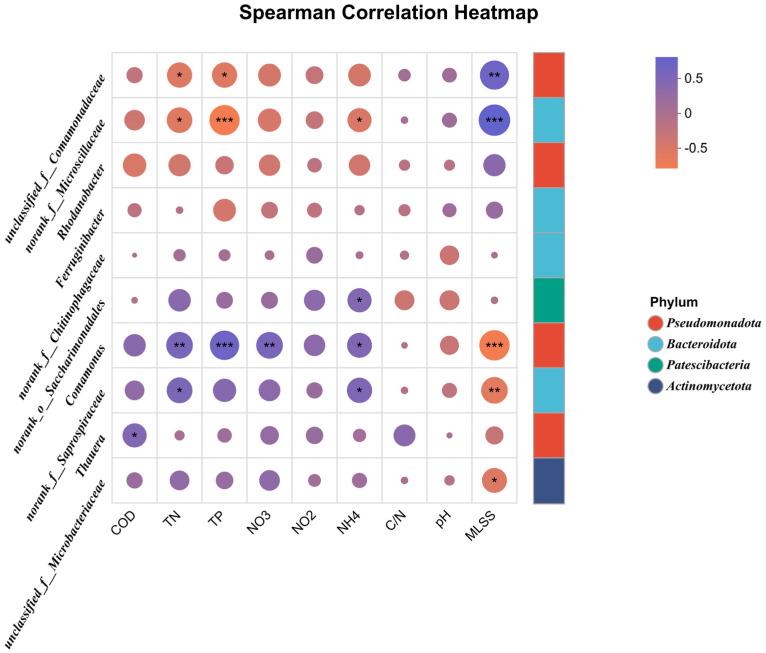
Correlation heatmap. *, ** and *** represent the level of correlation, *** being the strongest.

Based on Figure 11, I analyzed the potential correlations between bacterial genera and environmental factors. Correlation analysis revealed that nitrogen concentration and C/N ratio were the key environmental factors driving microbial community structure. *unclassified_f__Comamonadaceae* showed significant negative correlations with COD, TN, NO_3_^−^-N and NO_2_^−^-N, indicating its poor adaptability to the environment supplemented with the *Hordeum vulgare* var. *coeleste* straw carbon source. This genus is sensitive to high nitrogen substrate concentrations and cannot efficiently utilize straw-derived organic matter, leading to its reduced abundance in carbon source-added groups.

*Thauera* exhibited a significant positive correlation with TN and NO_3_^−^-N. As a core denitrifying bacterium, it can utilize various carbon sources including aromatic compounds abundant in straw hydrolysate for denitrification. According to relevant literature, strains of the genus *Thauera* can perform denitrification using various carbon sources including aromatic compounds [38]. However, it also showed certain maladaptation to the external carbon source environment, possibly due to intermediate metabolite stress or interspecific niche competition.

*Comamonas* and *norank_f__Saprospiraceae* showed extremely significant positive correlations with TN and C/N ratio, confirming their high utilization efficiency of the straw carbon source [30,31,32]. These two genera synergistically complete nitrogen removal through heterotrophic denitrification and have become the dominant nitrogen-removing bacteria in carbon source-supplemented systems.

Notably, almost all functional genera showed consistent correlations with TN, indicating that nitrogen concentration directly determines the abundance and distribution of nitrogen-removing flora. The divergent responses of different genera to C/N ratio further demonstrated that C/N ratio acts as a critical threshold factor driving microbial niche differentiation after straw carbon source addition. High MLSS creates a suitable metabolic microenvironment within sludge flocs for nitrogen-removing functional bacteria [3].

**Figure 12:**
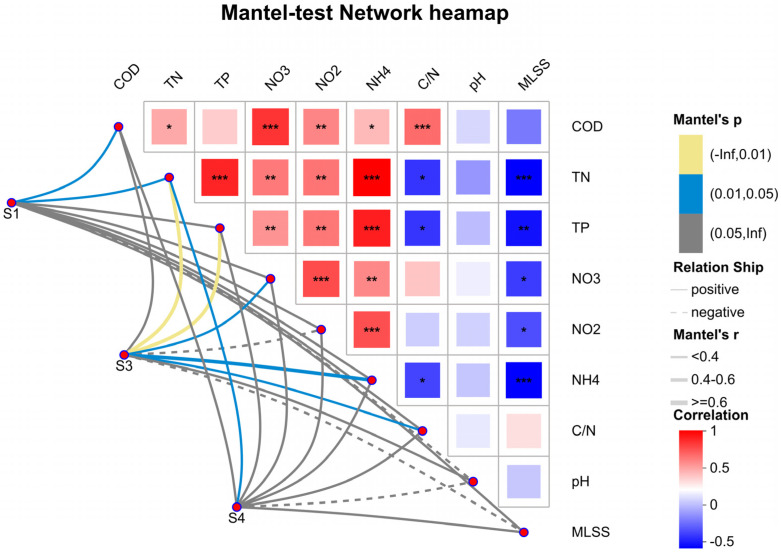
Mantel test network heatmap. *, ** and *** represent the level of correlation, *** being the strongest.

According to the Mantel test results (Figure 12), COD, TN, TP, NO_3_^−^-N, NO_2_^−^-N and C/N ratio exhibited extensive and highly significant positive correlations, forming the core environmental gradient that drove synchronous variations in nutrients in the system. In contrast, TN and NO_3_^−^-N showed significant negative correlations with MLSS, reflecting the efficient nutrient consumption by microbial communities during sludge enrichment. pH had no significant correlations with most nutrient indices, indicating it was not a core driving factor, and its stable range further confirmed the environmental stability of the system with straw carbon source addition.

Network analysis further revealed distinct environmental preferences of the three groups: S1 (blank control) and S3 (acid-pretreated) were mainly positively correlated with high nutrient loads (COD, TN, NO_3_^−^-N, and NO_2_^−^-N), reflecting their distribution in carbon–nitrogen-rich environments. S4 (alkali-pretreated) showed strong positive correlations with MLSS and C/N ratio, but negative correlations with nutrient indices, representing a stable stage with high sludge concentration, high C/N ratio and low nutrient levels. This indicates that alkali-pretreated *Hordeum vulgare* var. *coeleste* straw efficiently promoted microbial proliferation (elevated MLSS) and nutrient degradation, ultimately driving the system toward stable and efficient nutrient removal.

**Figure 13:**
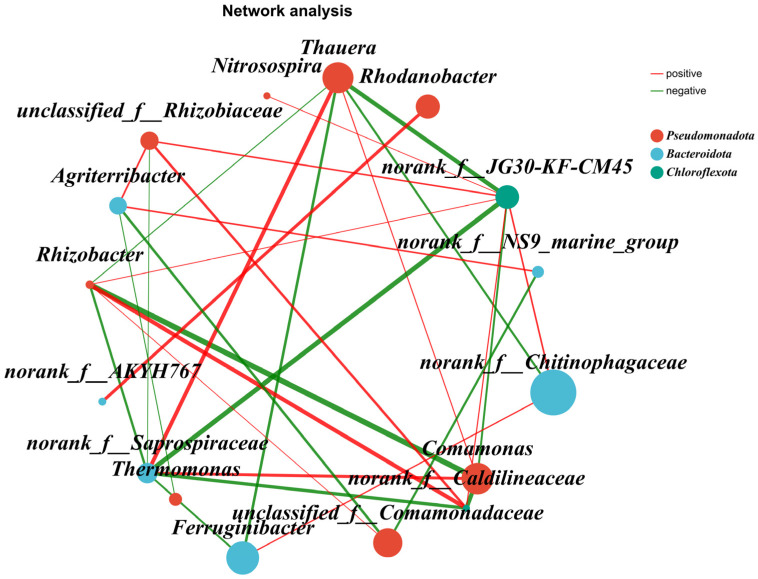
Species correlation network graph.

As can be seen from Figure 13, the top 20 genera in abundance mainly belong to three phyla: *Pseudomonadota*, *Bacteroidota*, and *Chloroflexota*. Among them, *Thauera*, *Comamonas*, and *unclassified_f__Comamonadaceae* are the dominant genera in *Pseudomonadota* [29,30,31,32,33,34,35,39]. *Thauera* and *norank_f__Saprospiraceae* exhibited promoting effects with strong positive correlations. *norank_f__Saprospiraceae* functions in organic matter degradation, which may complement the nitrification role of *Thauera*. There was a significant negative correlation between *norank_f__Saprospiraceae* and *norank_f__JG30-KF-CM45*, which may be related to environmental conditions. As mentioned earlier, *norank_f__JG30-KF-CM45* responded noticeably to the C/N ratio. *norank_f__Saprospiraceae* and *norank_f__JG30-KF-CM45* share similar functions and may thus compete with each other. Furthermore, *norank_f__Saprospiraceae* struggles to degrade *Hordeum vulgare* var. *coeleste* straw as a carbon source, whereas *norank_f__JG30-KF-CM45* can readily utilize small-molecule carbon sources from hydrolyzed *Hordeum vulgare* var. *coeleste* straw. *Comamonas*, a genus of *Pseudomonadota*, showed a significant negative correlation with *Rhizobacter*. It is inferred that these two genera overlap extensively in function, both performing denitrification and nitrogen removal, leading to competitive interactions. Additionally, the concentrations of their substrates, NO_3_^−^-N and NO_2_^−^-N, were low in water quality measurements. The shortage of substrates and functional overlap resulted in an inhibitory relationship between them. *norank_f__AKYH767* and *Rhodanobacter* showed no significant correlations with other genera in the figure, but a positive correlation between themselves. It is suggested that *norank_f__AKYH767* functions in the first step of short-cut denitrification, while *Rhodanobacter* functions in the second step. They act in a sequential upstream–downstream manner and complement each other in organic matter decomposition, thus showing a positive correlation.

**Figure 14:**
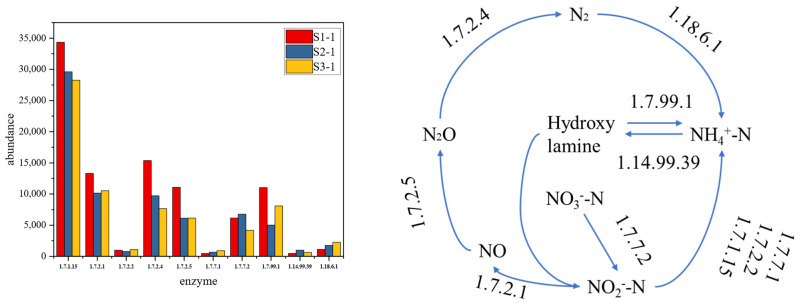

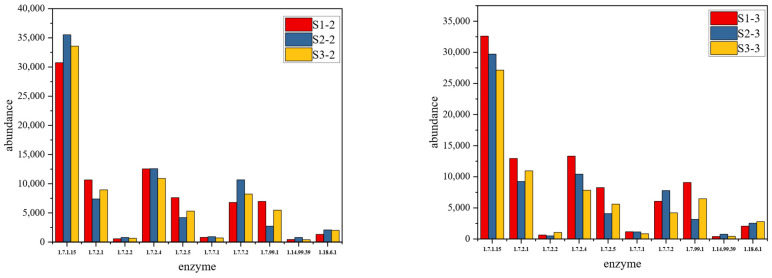
Metabolic pathway and abundance map of functional gene enzymes.

As can be seen from Figure 14, the roles and abundance changes in functional genes and enzymes involved in the nitrogen metabolism pathway are illustrated across various sub-pathways. Among them, the pathway with the highest abundance is the conversion from NO_2_^−^-N to NH_4_^+^-N. A comparison within the first control group reveals that the abundance of functional genes and enzymes in S1 is higher than that in S3 and S4. This indicates that the nitrogen removal efficiency with the addition of *Hordeum vulgare* var. *coeleste* straw as an external carbon source is superior to that of the control group without an external carbon source, and the high-abundance pathway reflects a significant advantage in denitrification. The second-most abundant pathway is the conversion from N_2_O to N_2_, followed by the conversion from NO to N_2_O, both of which are also denitrification processes. Moreover, S3 exhibits the highest abundance of functional genes and enzymes in nearly all pathways among the control groups, demonstrating that acid-pretreated *Hordeum vulgare* var. *coeleste* straw used as an external carbon source significantly improves nitrogen removal performance. Temporally, the abundance of functional genes and enzymes first increases and then decreases. In the first cycle, the abundance in S1-1 is notably higher than in the other two control groups. In the second cycle, the abundance in S1-2 decreases significantly, whereas that in S3 and S4 increases. This suggests that the addition of *Hordeum vulgare* var. *coeleste* straw as an external carbon source affects functional genes and enzymes: it promotes the increase in their abundance in microorganisms within a certain concentration range, but exhibits an obvious inhibitory effect when the concentration exceeds a threshold.

## 5. Conclusions

This study investigated the nitrogen removal performance and microbial mechanism of *Hordeum vulgare* var. *coeleste* straw as an external carbon source in AOA-SBR systems treating low C/N domestic sewage on the Qinghai–Tibet Plateau. The main conclusions are as follows:

1. The optimal influent C/N ratio for nitrogen removal was 7~9. At this ratio, the alkali-pretreated straw group achieved average NH_4_^+^-N and TN removal efficiencies of 94.46% and 61.32%, respectively, which were 41.66% and 98.83% higher than the blank control. Excessive carbon addition failed to further improve treatment performance and caused secondary COD pollution.
2. The 2% NaOH pretreatment was superior to 2% H_2_SO_4_ pretreatment, yielding higher carbon content (COD > 8000 mg·L^−1^), a higher proportion of rapidly biodegradable organic matter (≈30%), lower toxic inhibitory substances (≈10%), and a stable slightly alkaline environment (pH 7.5~8.0) that was optimal for denitrifying bacterial metabolism.
3. Straw carbon source addition reshaped the genus-level microbial community while preserving overall functional redundancy. Alpha diversity analysis showed that species richness decreased slightly in carbon-amended groups, with the acid-pretreated group exhibiting the most simplified community structure, while the alkali-pretreated group maintained higher species evenness and resilience. Beta diversity confirmed significant community differentiation among treatments, with TN, NH_4_^+^-N, NO_3_^−^-N and C/N ratio as the core environmental drivers. *norank_f__Chitinophagaceae* (specialized in plant polysaccharide degradation) became the absolute dominant genus, and its abundance was significantly positively correlated with C/N ratio. *Comamonas* and *norank_f__Saprospiraceae* were strongly positively correlated with TN and MLSS, synergistically completing heterotrophic denitrification. In contrast, the conventional denitrifier *Thauera* showed no significant response to C/N changes, and *unclassified_f__Comamonadaceae* was negatively correlated with nutrient concentrations, indicating that straw carbon sources primarily enriched genera with specific hydrolysate-degrading functions rather than traditional denitrifiers. C/N ratio acted as a key threshold factor, distinguishing microbial functional groups by showing opposite correlations with *Comamonas* (negative) and *norank_f__JG30-KF-CM45* (positive). Functional gene analysis revealed that denitrification pathway enzyme abundances first increased and then decreased with rising C/N, consistent with the nitrogen removal performance trend.
4. *Hordeum vulgare* var. *coeleste* straw is a cost-effective alternative carbon source, with acid-pretreated straw replacing 60% of sodium acetate and alkali-pretreated straw having a higher substitution rate. Further studies are required to verify its long-term performance under actual plateau low temperatures (5~15 °C) and full-scale engineering conditions.

## Figures and Tables

**Table 1:** Average removal rate.

Average Removal Efficiency	C/N = 5~7				C/N = 7~9				C/N = 9~11			
	S1	S2	S3	S4	S1	S2	S3	S4	S1	S2	S3	S4
COD	44.37%	52.12%	70.58%	71.43%	32.04%	73.46%	83.34%	74.89%	38.91%	66.28%	79.79%	80.38%
NH4^+^-N	68.22%	78.63%	88.25%	90.06%	52.80%	83.41%	90.35%	94.46%	74.42%	77.21%	78.35%	82.36%
TN	28.83%	35.83%	46.35%	47.91%	30.84%	49.36%	58.46%	61.32%	31.12%	33.04%	41.14%	52.28%

**Table 2:** GC-MS analysis of carbon source components in fermentation broth.

Retention Time	Compound	Relative Peak Area (%)	Retention Time	Compound	Relative Peak Area (%)	Retention Time	Compound	Relative Peak Area (%)
9.546	3-Heptanone, 6-methyl-	10.77	8.483	Benzaldehyde	8.26	5.888	2-Heptanone	17.04
0.842	l-Alanine ethylamide, (S)-	7.91	3.049	Hexanal	7.28	31.906	Pentadecanone, 6,10,14-trimethy	11.76
14.751	Benzene, 1,2,3,4-tetramethyl-	3.72	14.740	Benzene, 1,2,3,4-tetramethyl-	4.46	9.759	2-Octanone	4.54
16.011	Benzene, 1,2,4,5-tetramethyl-	3.55	16.004	Benzene, 1,2,4,5-tetramethyl-	3.62	14.758	Benzene, 1,2,3,4-tetramethyl-	4.41
1.171	2,3-Butanedione	2.41	14.573	Benzene, 1,2,4,5-tetramethyl-	2.02	9.638	5-Hepten-2-one, 6-methyl-	3.45
3.861	Butanal, 2-ethyl-3-methyl-	1.79	22.530	1-Hexylbicyclo [2.2.2]octane	1.65	16.019	Benzene, 1,2,4,5-tetramethyl-	2.98
13.972	2-Nonanone	1.75	1.724	Pentanal	1.36	9.579	3-Octanone	2.31
14.575	Benzene, 1,2,4,5-tetramethyl-	1.55	32.891	E-3-Pentadecen-2-ol	1.19	14.580	Benzene, 1,2,3,4-tetramethyl-	1.47

## Data Availability

The water quality data supporting this paper were obtained from the Xizang Civil Engineering, Water Conservancy and Electric Power Engineering Technology Research Center, and the microbial data were obtained after detection by Majorbio Bio-Pharm Technology Co., Ltd. The original contributions presented in this study are included in the article. Further inquiries can be directed to the corresponding author.
